# Insights into Trace Metal Metabolism in Health and Disease from PET: “PET Metallomics”

**DOI:** 10.2967/jnumed.118.212803

**Published:** 2018-09

**Authors:** Joanna J. Bartnicka, Philip J. Blower

**Affiliations:** King’s College London, School of Biomedical Engineering and Imaging Sciences, St. Thomas’ Hospital, London, United Kingdom

**Keywords:** copper trafficking, positron emission tomography, ^64^Cu, metallomics

## Abstract

Essential trace metals such as copper, zinc, iron, and manganese perform critical functions in cellular and physiologic processes including catalytic, regulatory, and signaling roles. Disturbed metal homeostasis is associated with the pathogenesis of diseases such as dementia, cancer, and inherited metabolic abnormalities. Intracellular pathways involving essential metals have been extensively studied but whole-body fluxes and transport between different compartments remain poorly understood. The growing availability of PET scanners and positron-emitting isotopes of key essential metals, particularly ^64^Cu, ^63^Zn, and ^52^Mn, provide new tools with which to study these processes in vivo. This review highlights opportunities that now present themselves, exemplified by studies of copper metabolism that are in the vanguard of a new research front in molecular imaging: “PET metallomics.”

Essential trace metals are important in many cellular and physiologic processes, including ubiquitous enzymatic reactions and protein folding. For example, copper-dependent enzymes include cytochrome-c oxidase, superoxide dismutase, and ceruloplasmin, which participate in aerobic respiration, antioxidant defense, and hepatic iron release, respectively, all of which depend on the redox activity of copper as it cycles between its 2 principal oxidation states, Cu(I) and Cu(II). Zinc is essential for the catalytic function and structure of many proteins with roles in, inter alia, metabolism, gene regulation, chromatin structure, and neurotransmission. Iron is essential in hemoglobin, respiration, and DNA and RNA synthesis, whereas manganese is important to enzymes that destroy reactive oxygen species and produce urea and nitric oxide (1). In healthy states, metal homeostasis is tightly controlled, and its deregulation is implicated as cause or consequence of many human diseases. Mutations in copper transporters ATP7B and ATP7A result in, respectively, Wilson’s disease (WD) (characterized by copper overload) and Menkes disease (characterized by systemic copper deficiency and fatal in childhood). Copper imbalance is implicated in neurodegeneration and cancer, although whether as cause or consequence is unclear. Abnormal zinc levels occur in diabetes and cystic fibrosis, and both zinc and copper accumulate in amyloid plaques in Alzheimer disease. The study of trace metals in biology and medicine, historically known as *inorganic biochemistry* or *bioinorganic chemistry*, is growing in importance, becoming fashionably known as “metallomics.”

The structure and function of many metalloproteins containing copper, zinc, and other trace metals are well characterized. The routes through which these metals reach their sites of action and become incorporated into their metalloproteins are less well understood, but it is now clear that complex trafficking systems exist in cells to ensure correct delivery while avoiding toxic accumulation. Metal concentration in tissues can be measured and mapped by highly sensitive techniques such as laser ablation inductively coupled plasma mass spectrometry, but transport and chaperoning pathways leading to these local concentrations—from ingestion to site of action and excretion—are less well studied. Metal ion–activated optical (2) and MRI (3) probes for cellular and in vivo whole-body measurement of copper and zinc have been described. However, these probes must react with the metal ions; hence, they visualize exchangeable copper pools rather than copper trafficking processes, and may themselves perturb the systems they are probing. Dynamic, acute metal fluxes in the whole body that give rise chronically to the endpoints represented by total copper content of tissues are not amenable to study in vitro at the cellular level. Practitioners of molecular imaging and nuclear medicine will instinctively recognize these whole-body trafficking processes as precisely those that their methods and instruments can help elucidate.

## UTILITY OF PET IMAGING TO MONITOR METAL HOMEOSTASIS

**FIGURE 1.**
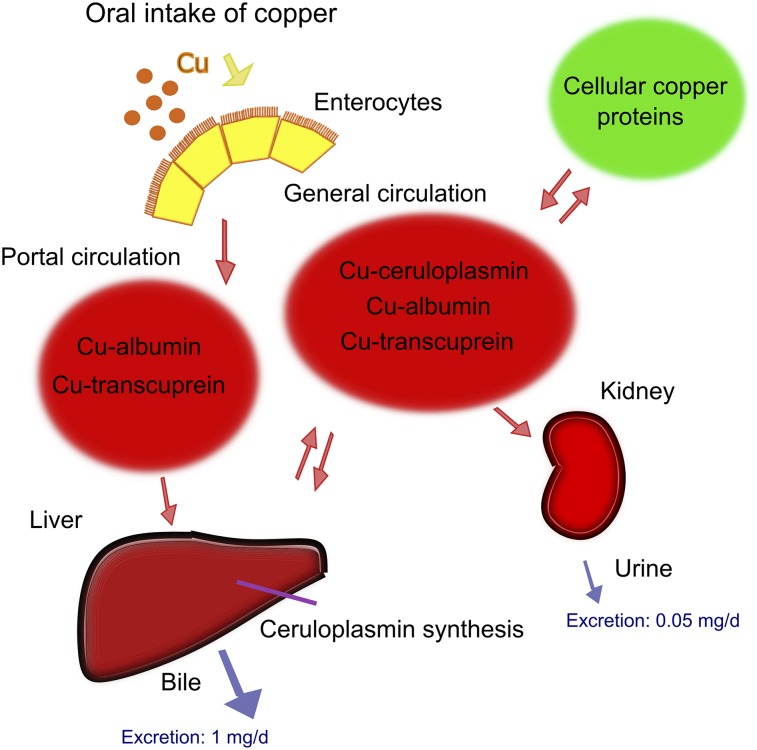
Simplified model of whole-body copper absorption, distribution and excretion, showing main identified copper carriers in blood. Based on data from Linder (40).

Radioactive and stable metal isotopes have been used historically, without the advantages afforded by radionuclide imaging, to delineate trafficking pathways between body compartments for metals such as copper (Fig. 1). However, the need for tissue sampling restricted their use in humans and required culling of animals at predetermined time points. In vivo imaging with PET circumvents these problems, allowing noninvasive, dynamic measurements across the whole body. With growing availability of PET scanners and rapidly developing methodology for producing positron-emitting radioisotopes of key essential metals, it is now increasingly feasible to use nuclear medicine techniques to address these challenges. Although this use of PET is in its infancy, recent studies with ^64^Cu exemplify the potential, marking a path that can be followed to study other essential trace metals.

## PET IMAGING OF COPPER HOMEOSTASIS

^64^Cu has been routinely available in some research-oriented PET centers for more than two decades. Chelator design for ^64^Cu radiolabeling of biomolecules and redox-active tracers is advanced (4), but use of ^64^Cu to study the biologic behavior of copper itself has been relatively scarce. ^64^Cu decays by 18% β^+^ emission with a half-life of 12.7 h, allowing imaging for days, long enough to image both uptake in tissues and subsequent efflux and excretion (unlike other positron-emitting copper radioisotopes: ^60^Cu, ^61^Cu, ^62^Cu). The small but growing literature describing its use in metallomics in health and in disease, particularly WD, dementia, and cancer, are reviewed below.

### WD

WD is caused by an autosomal recessive mutation in the ATP7B gene. Loss of function of ATP7B disables copper incorporation into ceruloplasmin and excretion via bile. The resulting systemic copper overload causes liver dysfunction, severe neurologic symptoms, and eye and kidney damage. Radiocopper has historically been used to detect differences in copper metabolism between WD patients and healthy individuals by measuring radioactivity in serum and excreta. More recently, PET in mice 24 h after intravenous or oral administration of ^64^CuCl_2_ showed that ATP7B knockout increased tracer liver uptake severalfold and decreased its biliary excretion, agreeing with clinical manifestations of WD (5). Because many WD patients present with neurologic or psychiatric symptoms, possibly linked to disrupted cerebral copper homeostasis, ^64^CuCl_2_ PET has been used to image brain copper fluxes in ATP7B knockout mice (6), revealing significant age-dependent differences from healthy controls. However, brain uptake of ^64^Cu administered as CuCl_2_ is so low that interpreting these changes is difficult. The acute changes observed by PET did not correlate with total brain copper content measured by inductively coupled plasma mass spectrometry, demonstrating the need for complementary information from both methodologies to fully understand acute and chronic effects of disease on whole-body copper homeostasis. Nevertheless, these animal studies suggest that ^64^Cu PET could become a clinical tool to assess whole-body copper metabolism in humans to improve patient management and reduce need for invasive liver biopsy. It could complement genetic testing (which is challenging to interpret as more than 300 disease-causing mutations of ATP7B have been identified (7)) and monitor outcomes of treatments such as copper chelation, gene therapy, and hepatocyte transplantation. PET imaging with ^64^Cu-histidine complex has been used in the rat model of WD in the latter context (8).

### Neurodegeneration

**FIGURE 2.**
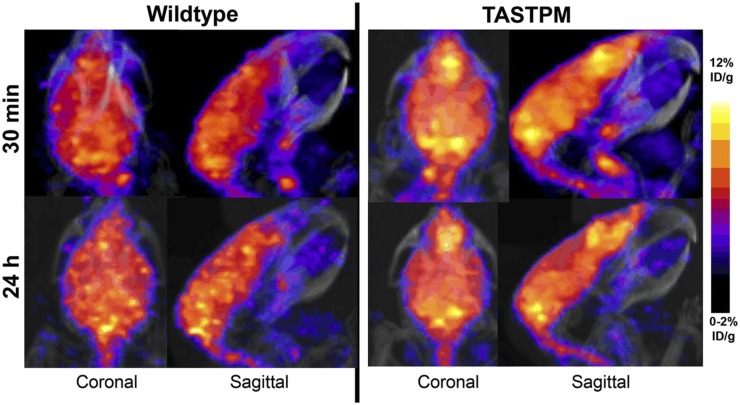
PET/CT maximum-intensity-projection images showing differences in the regional brain uptake of ^64^Cu in wild-type and TASTPM AD model mice, 30 min and 24 h after intravenous injection of ^64^Cu-GTSM. (Reprinted with permission of (12).)

Growing evidence implicates zinc, iron, and copper in the pathology of β-amyloid aggregation associated with Alzheimer disease (AD)—the “metal hypothesis of AD” (9). The role of these metals, whether cause, consequence, or merely red herring, is poorly understood, and could potentially be illuminated by imaging brain metal fluxes. Peng et al. found no significant age-dependent changes in brain copper uptake by ^64^CuCl_2_ PET in healthy mice (10), again emphasizing the limitations of ^64^CuCl_2_ PET in brain due to low blood–brain barrier penetration. To circumvent this problem, others used ^64^Cu-GTSM, a lipophilic complex that crosses the blood–brain barrier and passively enters cells, by-passing copper trafficking mechanisms and barriers. It then undergoes reductive dissociation to liberate ^64^Cu^+^, which, it is presumed, feeds into intracellular copper trafficking pathways and can be used to observe copper redistribution and efflux. ^64^Cu-GTSM PET and ex vivo quantification in a mouse model of AD, βPP/PS1 (11), showed significantly higher uptake in the brains of the AD mice than in controls (*P* = 0.01) after 1 h. A similar ^64^Cu-GTSM study (12) revealed an analogous difference in another AD model, TASTPM transgenic mice, showing also that ^64^Cu clearance from the brain between 30 min and 24 h was faster, and heterogeneity of cerebral distribution was greater, than in wild-type controls (Fig. 2). Interestingly, both groups showed no correlation in regional distribution between ^64^Cu uptake and Aβ plaques. Use of ^64^Cu-GTSM highlights the importance of evaluating alternatives to simple radiometal salts, intravenous injection of which may not mimic native pathways and which cannot visualize regions of low uptake, such as the brain, in which uptake of ^64^Cu-acetate was more than 7-fold lower than that of ^64^Cu-GTSM and was limited to the ventricular areas (13).

### Cancer

Copper metabolism is disrupted in various human cancers. Plasma copper levels are elevated in some cancer patients, possibly correlating with cancer progression (14), and increased copper concentrations are found in tumor tissue. Copper is an essential cofactor for proteins involved in tumorigenesis and can modulate cancer cell proliferation, angiogenesis, and metastatic progression in vitro. Pharmacologic manipulations of a “copper-cancer axis” showed cytostastic effects in preclinical models (15). Mouse hepatomas accumulated considerable ^64^Cu (16) on administration of radiocopper(II) salts. The study of copper trafficking in cancer is therefore worthwhile, particularly after identification of the human high-affinity copper transporter 1 (hCTR1). PET with intravenous ^64^CuCl_2_ was evaluated in athymic mice bearing hepatoma xenografts, with hCTR1 as its putative molecular target, and later in mice bearing various human cancer xenografts including prostate, hepatocellular carcinoma, melanoma, head and neck, glioblastoma, and breast cancers (17,18), showing promise particularly in anatomic regions with low background ^64^CuCl_2_ uptake such as the pelvic area and brain. Some of these studies confirmed higher hCTR1 expression in malignant cells than in nonmalignant or background tissue. Although 1 study in 5 cancer cell lines found no correlation of ^64^CuCl_2_ uptake with hCTR1 expression (measured by quantitative polymerase chain reaction) (19), others showed that hCTR1 knockdown in PC3 prostate cancer xenografts reduced ^64^Cu uptake from 7.21 to 4.02 percentage injected dose per gram (*P* < 0.001) (20) and hCTR1 overexpression in a MDA-MB-231 breast cancer model increased it from 2.58 to 5.37 percentage injected dose per gram (*P* < 0.05) (21). These observations suggest that hCTR1 is important, but not the sole player, in the in vivo tumor uptake of copper.

### Clinical Studies in Cancer Patients

**FIGURE 3.**
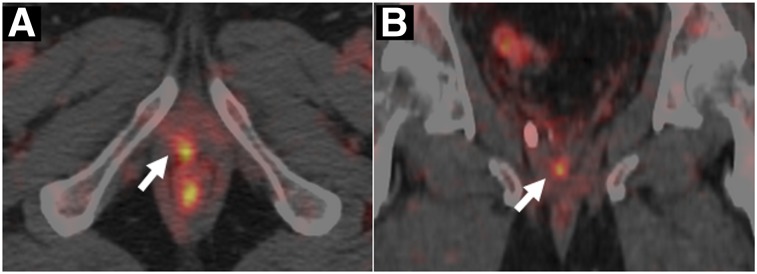
Axial (A) and coronal (B) PET/CT images of pelvic area in prostate cancer patient (Gleason grade 4 + 3) 60 min after injection of 200– 250 MBq of ^64^CuCl_2_, demonstrating tracer uptake in foci of local relapse (arrow). (Reprinted with permission of (24).)

^64^CuCl_2_ PET has recently been evaluated for tumor imaging in humans, detecting primary and secondary lesions in 18 of 19 patients with glioblastoma multiforme (22) (although uptake may have been due to blood–brain barrier disruption rather than specific copper transport pathways). In prostate cancer, the low urinary excretion of ^64^CuCl_2_ (23) allowed local tumors to be readily delineated by PET in a preliminary study of 7 patients. A larger study in 50 prostate cancer patients (24) demonstrated detection of tumor recurrence and lymph node metastases (Fig. 3) with greater sensitivity than ^18^F-choline, especially in patients with low prostate-specific antigen levels. Further trials of ^64^CuCl_2_ PET comparing it with established prostate cancer tracers, and studies of the specific pathways involved, are warranted and encouraged by lack of observed adverse effects of the ^64^CuCl_2_ doses (200–250 MBq) used. Dosimetry estimates showed that several GBq could be administered before exceeding threshold radiation doses to the liver (the critical organ) (23–25).

^64^Cu PET visualization of copper homeostasis in tumors could aid development of new treatment strategies, exploiting the putative roles of copper in carcinogenesis. Despite encouraging preclinical results, clinical trials involving cancer treatment with copper chelators, or combining copper supplementation with ionophores, mostly failed to demonstrate clinical efficacy (15). Patient stratification using ^64^Cu PET to visualize abnormal copper homeostasis may improve the sensitivity of such trials (26).

The combined β, positron, and Auger electron emissions of ^64^Cu offer applications in radionuclide therapy, which could be further supported by growing availability of the longer half-life β-emitter ^67^Cu. Despite promising tumor reductions in mice bearing xenografts of human melanoma (27) and glioblastoma (28), evidence of therapeutic effectiveness of ^64^CuCl_2_ in human subjects is sparse (29). Dosimetric analysis in the prostate cancer trial (25) suggested that tumor absorbed doses from ^64^Cu would be too low for therapeutic benefit, but the analysis did not include effects of Auger electrons and was not extended to ^67^Cu.

## PERSPECTIVE

^64^CuCl_2_ PET is increasingly being used to assess whole-body copper fluxes in disease states caused by, or linked to, altered copper homeostasis, potentially leading to improved diagnosis and complementing or replacing biopsies (which are invasive and prone to anatomically incorrect sampling) and genetic testing (which is challenging in diseases with high genotypic variation). This field is immature, and experimental design and data interpretation are understandably naïve. Data on real-time trafficking of copper must be complemented by measurements of the consequent total copper content in tissues over long periods; it is possible that large differences in total copper content between tissues could arise from very small, perhaps undetectable, differences in acute trafficking. It is also clear that intravenous administration of ionic ^64^Cu (e.g., copper(II) chloride or acetate) alone will not provide full understanding of copper trafficking mechanisms as it probably does not reflect native trafficking pathways. Imaginative variations in the chemical form and route of administration must be devised.

Progress made in ^64^Cu PET is attributable to the established production methods and favorable imaging properties of ^64^Cu, and the proliferation of preclinical PET scanners. New radionuclide production methods are now extending this opportunity to other essential metals, particularly zinc, manganese and iron. The first animal and human zinc radioisotope studies used ^65^Zn, which due to its long half-life (243.7 d) presents little potential for imaging. ^62^Zn (half-life, 9.2 h; 8.4% positron yield) decays to the positron-emitting copper radioisotope ^62^Cu (half-life, 9.7 min), so that most positrons detected in PET scans would be from the daughter ^62^Cu (Table 1), confounding image interpretation. A promising radioisotope without this problem is ^63^Zn (half-life, 38.47 min). De Grado et al. developed production of ^63^Zn-citrate by proton-irradiating isotopically enriched ^63^Cu (30), described its biodistribution in mice, and performed a preliminary clinical study with intravenous ^63^Zn-citrate in 6 healthy human subjects and 6 with AD (31), confirming high uptake in the liver and pancreas and low but detectable brain accumulation previously seen in animals. Efflux kinetics from brain regions showed significant differences between healthy and AD patients, inviting future research on imaging brain zinc homeostasis in neurodegeneration. The availability of ^63^Zn, albeit limited to locations with an on-site cyclotron, allows imaging of zinc trafficking in the many diseases in which zinc is implicated (e.g., diabetes, dementia, hypertension, and prostate cancer).

**TABLE 1:** PET Isotopes of Metals with Potential for Imaging Whole-Body Metal Trafficking

Isotope	Production	Half-life	β^+^ yield (%)	Mean β^+^ energy (MeV)	Daughter	Reference
^64^Cu	Cyclotron	12.7 h	17.4	0.655	^64^Ni (stable)	5
^62^Cu	Generator	9.74 min	98	2.93	^62^Ni (stable)	NA
^62^Zn	Cyclotron	9.26 h	7.6	0.259	^62^Cu (β^+^)	NA
^63^Zn	Cyclotron	38.47 min	92.7	0.992	^63^Cu (stable)	30
^65^Zn	Cyclotron	243.9 d	1.7	0.143	^65^Cu (stable)	NA
^52^Mn	Cyclotron	5.59 d	29.6	0.24	^52^Cr (stable)	32
^51^Mn	Cyclotron	46.2 min	97.1	0.96	^51^Cr (γ)	35
^52^Fe	Cyclotron	8.28 h	56.1	0.344	^52m^Mn (β^+^)	39

NA = not applicable.

The recent development of a simple cyclotron-based production of ^52^Mn (half-life, 5.59 d), hitherto used only for antibody (32), liposome, and cell (33) labeling, offers the possibility to study manganese biochemistry in vivo. Paramagnetic Mn^2+^, which has been used as MRI contrast to trace neuronal connection and repair, is limited by the high, potentially neurotoxic concentration required, but recently PET with ^52^Mn^2+^ was evaluated for similar purposes (34). Whole-body biodistribution of ^52^Mn and the shorter-lived ^51^Mn (half-life, 45.59 min), administered as MnCl_2_, has been described in rodents (32,34–36), showing high uptake in the pancreas, which differed between healthy, obese, and diabetic mouse models. This warrants further evaluation of radiomanganese PET for imaging functional β-cell mass (36).

^52^Fe (half-life, 8.28 h), ^59^Fe (half-life, 44.5 d), and ^55^Fe (half-life, 2.73 y) have historically (since the 1960s) been used to visualize bone marrow function in patients and to assess iron turnover and organ uptake (37). Of these radioisotopes, only ^52^Fe emits positrons, and interpretation of scans is complicated by the positron emission of the daughter ^52m^Mn. It has been used to track Fe-hydroxide polysaccharides by PET in animals and humans (38). ^52^Fe-citrate was used in a small clinical study to delineate differences in brain iron metabolism between healthy and WD patients (39).

The examples above highlight the opportunity to use trace metal PET probes to help understand the role of trace metals in health and disease, to diagnose disease and monitor therapy outcomes longitudinally. With appropriate animal models and choice of administered chemical form, this could lead to new therapeutic strategies and methods to assess their effectiveness in patients by observing their effects on metal fluxes in vivo. These early studies demonstrate the growing feasibility of using PET to visualize metal trafficking pathways in vivo at the whole-body level, opening new exciting research avenues in the emerging field of PET metallomics.

## DISCLOSURE

This study was supported by the Medical Research Council, Wellcome Trust, Cancer Research U.K. No other potential conflict of interest relevant to this article was reported.
